# A biofidelic mock residual limb for prosthetic socket testing

**DOI:** 10.33137/cpoj.v8i2.45759

**Published:** 2025-09-10

**Authors:** C Phillips, A Nagpal, F Azhari

**Affiliations:** 1 Department of Mechanical and Industrial Engineering, University of Toronto, Toronto, Canada.; 2 Division of Engineering Science, University of Toronto, Toronto, Canada.

**Keywords:** Prosthetic Sockets, Mock Residual Limb, Adjustable Sockets, Biofidelic, Bench-Top Evaluation, Limb Loss, Artificial Limbs, Volume Change, Transtibial, Rehabilitation, Amputation, Prosthesis

## Abstract

**BACKGROUND:**

Evaluating prosthetic socket fit and function relies on accurately simulating load transfer between the residual limb and the socket. This limb can be either real (of a study participant) or a mock residual limb that simulates in vivo properties. Mock limbs minimize reliance on resource-intensive clinical trials; however, most are static in size, limiting their use in testing clinical outcomes like socket adjustability.

**OBJECTIVE:**

To design and validate a biofidelic mock limb, capable of real-time, controllable volume adjustments of up to ±5% limb volume.

**METHODOLOGY:**

Water-filled bladders were embedded within a transtibial residual limb model made of a dual-durometer urethane composition, mimicking deep and soft tissue. An Arduino-controlled syringe system was used to actuate volume adjustments. The method was validated through repeatability tests at different rates of volume change, cycling through expansion, holding at maximum volume, and contraction. Volume change was quantified by measuring interfacial pressures between the limb and a static socket.

**FINDINGS:**

The limb was fabricated with readily available materials for less than CAD 400. Volume change rate had minimal effect on interfacial pressure throughout the testing cycle, and minimal hysteresis was found between expansion and contraction periods. Repeatability was high, with a coefficient of variation of normalized pressure remaining below 10.4% over three repeated tests.

**CONCLUSION:**

The proposed biofidelic limb was validated for its ability to mimic volume change in a transtibial residual limb. The design enables easy replication or customization to simulate different limb physiologies and anatomies. The limb allows for controllable bench-top testing during prototyping of adjustable sockets or other devices, thus bringing devices to clinical use sooner.

## INTRODUCTION

A prosthesis user's residual limb can fluctuate significantly in size due to factors like activity and diet.^1,2^ As a result, traditional rigid prosthetic sockets fail to maintain a proper coupling at the residuum-socket interface, which is essential for comfort, function, and the prevention of skin and soft tissue problems.^3,4^

Adjustable sockets are designed to accommodate an expanding or contracting limb via straps, movable panels, or other dynamic mechanisms.^5-7^ To evaluate their performance, testing protocols must involve a volume-fluctuating residual limb, which can either be a study participant's limb or a lab-fabricated mock limb. Clinical testing with study participants is time- and resource-intensive, requiring meaningful limb volume fluctuation and accurate measurement of that volume change within the socket. Thus, apart from when subjective feedback (e.g., comfort scores) is needed, using a biofidelic mock limb—engineered to replicate both the material composition and volume fluctuations of a physiological limb—offers a more practical, cost-effective alternative.

Various mock limbs are used today, each designed to meet specific testing goals. Rigid limbs may suit structural testing of sockets to failure, as described in recent reviews^8-10^ and recommended by ISO 10328,^11^ whereas soft, compliant limbs may better replicate biomechanics at the residuum-socket interface, allowing for more precise evaluation of tissue strains^12,13^ and suspension effectiveness.^14^

Most prior mock limbs for testing adjustable sockets have been static, necessitating different mock limb sizes to simulate varying volumes.^6^ Seo et al.^2^ used five mock limbs of different volumes (neutral, ±3%, ±7%) to test their adjustable socket, and Murdoch^15^ used three mock limbs to investigate socket fit across three limb volumes (neutral and ±10%). This approach limits the ability to study how the socket responds to a specific rate of limb volume change in real time. One exception to a static mock limb is a “residual limb simulator” used by Paterno et al.^16^ to test the functionality of a flexible transfemoral prosthetic socket. The simulator utilizes a controllable syringe pump (similar to the design in this paper) to increase limb volume; however, no description of its materials, design or fabrication is provided.

In this paper, the design and fabrication of a ‘biofidelic’ mock residual limb, capable of real-time, controllable volume adjustability, is presented. To validate the design, trends in interfacial pressure changes (between the limb and a rigid socket) were analyzed to assess repeatability at different rates of volume change, and the symmetry of volume change between two localized regions of the biofidelic limb was also examined.

## METHODOLOGY

### Design Criteria and Requirements

To determine specific and meaningful design criteria, focus was placed on modeling a transtibial residual limb. The design choices were: region of adjustability, extent of adjustability, rate of volume adjustment, and material composition.

Residual limb volume changes typically occur radially (rather than axially),^6^ and are localized to regions with more interstitial fluid, thus away from bony prominences.^17,18^ A biofidelic mock limb with localized, radial volume adjustability was designed to reflect this. While the design approach allowed for bladder size and placement customization, for this study locations were selected based on literature describing pressure-tolerant areas typically actuated by panel-based adjustable sockets: the medial and lateral tibial flare regions and the posterior compartment distal to the popliteal.^6,19,20^ A circumferentially symmetric configuration of three adjustable regions was selected to simulate these anatomical areas and to facilitate symmetry verification during expansion and contraction.

Socket fit is often adjusted by adding sock plies, with over 5-ply considered clinically unacceptable,^21^ and 10-ply warranting a new socket.^22^ For a limb of 6 cm radius, a 5-ply sock (1.2 mm thick) corresponds to a 4% volume increase. Therefore, we targeted a ±5% volume change.

Volume change rates in a mature residual limb vary widely depending on factors such as activity level and diet, and have been shown to range from as slow as ±0.07%/min to ±3.3%/min after various activity programs.^23^ To capture this variability, testing was conducted across three orders of magnitude: from ±0.01%/min to ±10%/min.

The biofidelic mock limb consists of a dual-durometer urethane bulk, surrounding a rigid centre mandrel. This composition allows for an effective force transfer from a mechanical load frame, while also offering adequate compliance at the limb-socket interface to simulate the relative movement of the limb within the socket,^24^ which is an important outcome measure of adjustable sockets. Water-filled bladders were used to adjust volume. Water was chosen over air for its incompressibility, ease of use, and similar material properties to physiological interstitial fluid or blood.^17^

### Design Overview and Fabrication

**Figure 1:**
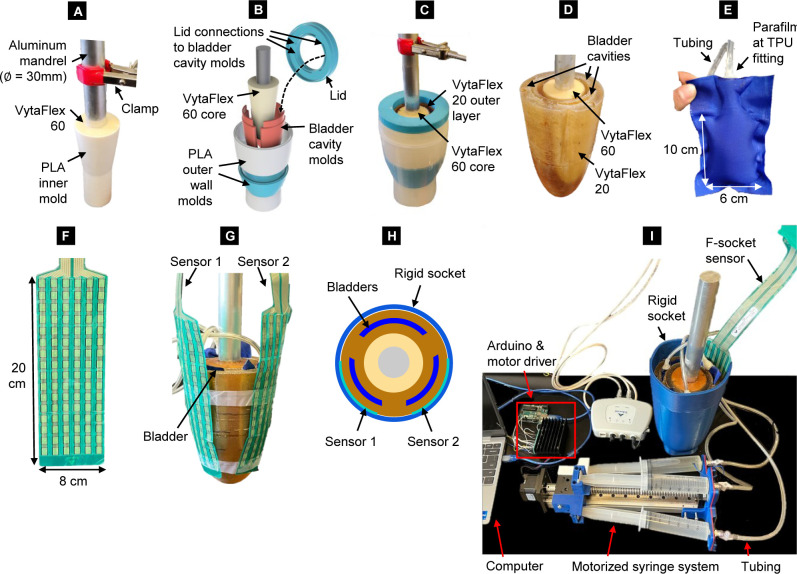
Design overview. **A**) Step one: casting the core with VytaFlex™ 60 around an aluminum mandrel. **B**) Mold set-up for casting the outer layer. The bladder cavity molds are held in place by clipping into the lid. The wall molds were separated to ease mold removal post-curing. **C**) Step two: casting the outer layer with VytaFlex™ 20. **D**) Final casted limb. **E**) TPU bladder. **F**) F-socket sensor, 0.15 mm thin. **G**) Sensor placement on the limb before donning the rigid socket. **H**) Top view schematic of sensor placement on the limb. **I**) Full system with the biofidelic mock limb, rigid socket, and motorized syringe system. Only one F-socket sensor is shown for clarity.

The final design comprises three water-filled bladders embedded in a dual-durometer urethane mock limb. Inspired by fabrication methods described by Quinlan et al.^24^ for a static dual-durometer urethane limb, firstly, the inner core of the limb was casted with VytaFlex™ 60 (shore A hardness, Smooth-On, Reynolds Advanced Materials, Chicago, IL) around an aluminum mandrel (elastic modulus = 70 GPa) suspended approximately 2 cm from the mold bottom using a clamp stand during the 24-hour casting (**Figure 1A**). Next, a custom mold assembly was designed to form the bladder cavities within an outer layer of VytaFlex™ 20 (shore A hardness 20, Smooth-On, Reynolds Advanced Materials, Chicago, IL) (**Figure 1B and 1C**). The cured inner core was suspended within the outer socket shell approximately 3 cm from the mold bottom using the clamp stand during casting of the VytaFlex™ 20 (24 hours). Lastly, molds were removed, leaving the final dual-durometer urethane limb (**Figure 1D**) with a volume of 1,330 mL at its fully-contracted state. All molds were 3D printed in PLA, sealed to a smooth finish with a thin layer of epoxy, and coated with two layers of Universal™ Mold Release (Smooth-On, Reynolds Advanced Materials, Chicago, IL).

TPU fabric was heat-sealed to form 6 x 10 cm bladders, which were manually inserted into the cavities of the cured VytaFlex™ 20. Each bladder was heat-sealed to a 3D printed TPU fitting made for 6.4 mm tubing. Parafilm secured the seal at the tube fitting (**Figure 1E**). Preliminary testing showed each bladder could hold 60 mL of water without leaking. Given a fully-contracted limb volume of 1,330 mL, full bladder inflation would theoretically yield ±6.8% volume adjustability, meeting design requirements. Bladders were connected to a motorized syringe system, controlled using a linear actuator and Arduino microcontroller. The motor offered a torque of 1.9 Nm, and power of 36 W per rotation. From preliminary testing, each syringe was expected to withstand a maximum fluid pressure of 200–250 kPa from the bladders at full capacity. To reduce costs, all three syringes were controlled with a single motor. If desired, each syringe can be controlled by its own motor. The total cost for all parts is less than CAD 400. Parametric design files of the molds, a Bill of Materials and Arduino code are available on GitHub: https://github.com/Decisionics/BiofidelicLimb.

For performance evaluation, the limb was placed in a rigid 3D printed PLA socket (5 mm wall thickness). The socket's inner geometry matched that of the fully-contracted limb. Bladders were then expanded and contracted at various rates. Two F-Socket™ sensors (**Figure 1F**) from VersaTek (Tekscan Inc., South Boston, MA) were placed between the limb and socket at two bladder locations (**Figure 1G and 1H**) to read interfacial pressure. A maximum of two sensors could be used simultaneously with our 2-Cuff F-Socket™ VersaTek system (Tekscan Inc., South Boston, MA). At full contraction, an average pressure on the limb of 3–5 kPa was read, which was deemed suitable for a loosely fitted socket.^25^ The full system set-up is shown in **Figure 1I**.

### Protocol for Evaluating Adjustability

**Figure 2:**
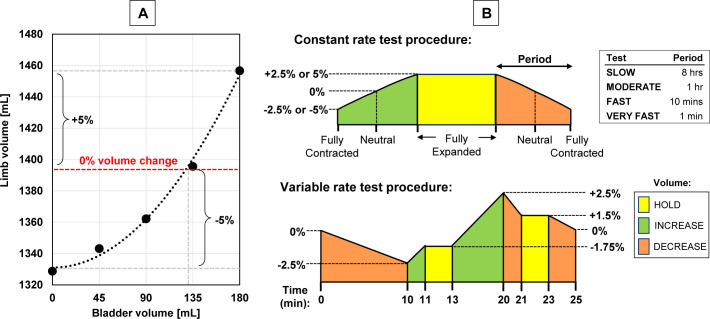
**A**) Relation between limb volume [mL] and bladder volume [mL] to measure the bladder volume required to obtain a ±5% limb volume change. **B**) Testing protocol (limb volume change [%] vs. time) for constant (top) and variable (bottom) rates of change test procedures.

The bladders theoretically allow for a ±6.8% volume change; however, this may not directly reflect total limb volume change since bladders may, to some extent, compress surrounding urethane without significantly increasing overall limb size. To quantify the relation between bladder volume and limb volume, limb volume was measured at bladder volumes from 0–180 mL using circumferential measurements taken at 2.5 cm intervals along the length of the limb. As shown in **Figure 2A**, bladders must be fully expanded to achieve the desired ±5% limb volume change.

The biofidelic limb was subjected to four different volume change rates inside the socket: **1)** a SLOW rate over 24 hours, **2)** MODERATE rate over 3 hours, **3)** FAST rate over 30 minutes, and **4)** VERY FAST rate over 3 minutes. Tests started with the limb at its fully contracted size, then volume increased to its fully-expanded size, was held, then contracted back to its original volume (**Figure 2B**). The limb's behaviour (detected by measuring interfacial pressures) across different total volume change amounts was assessed by conducting the SLOW (0.01%/min), MODERATE (0.08%/min), FAST (0.5%/min) and VERY FAST (5%/min) tests for volume changes between -2.5% and +2.5%. Volume changes of ±5% were also tested; however, due to resource constraints of the study, this was limited to FAST and VERY FAST rates conducted at double speed (1%/min and 10%/min, respectively). Furthermore, a variable-rate protocol was tested that cycled the limb through different volume change rates (**Figure 2B**). This assessed the system's ability to vary both the rate and direction of syringe motion in a non-sequential manner, simulating potential complex volume change patterns that might occur over periods of time involving a combination of activities such as walking, sitting and standing.^1,19,23^ Tests were repeated three times. Coefficient of variation (CV = standard deviation/mean × 100%) was calculated to quantify repeatability among volume change rates, among repeat tests at the same volume change rate, and between the two sensors to quantify symmetry of volume change at two bladder locations.

### Interpreting the F-Socket Pressure Readings

The F-Socket sensors are marketed for one-time use, and experience significant drift over a period of 20 minutes.^26-28^ However, due to the high cost of each sensor, only one set of sensors was used for all tests, in which the sensors were loaded over three days. Precise pressure magnitudes were not necessary in this study; instead, analyzing patterns of pressure changes sufficed to evaluate the functionality of the limb through metrics like repeatability, hysteresis, and symmetry. Therefore, results are normalized to the maximum pressure reached in each test to allow for comparison between tests. Each pressure recording is an average reading across all sensels of the sensor in contact with the bladder.

## RESULTS

### Effect of Volume Flow Rate on Pressure Distribution

**Figure 3:**
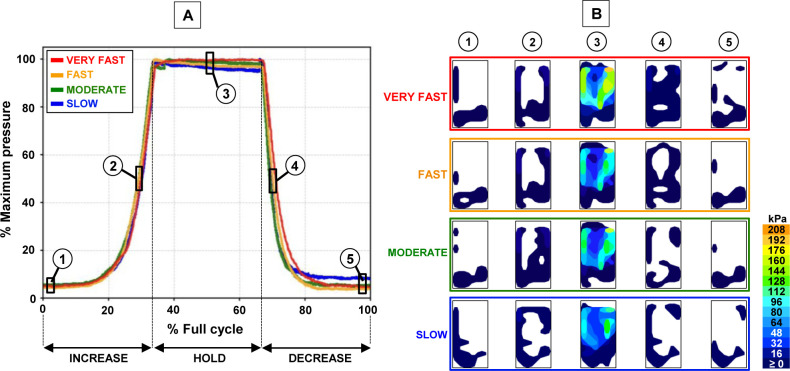
**A**) Normalized pressure [%] vs. cycle time [% full cycle] for different volume flow rates, measured from one F-socket sensor. **B**) Pressure distribution maps shown at (**1**) -2.5% and (**2**) 0% during expansion, (**3**) +2.5%, then (**4**) 0% and (**5**) -2.5% during contraction.

Volume flow rate had little effect on interfacial pressure distribution and rate of pressure change across all testing cycles. **Figure 3A** shows similar trends in normalized pressure for ±2.5% volume change, regardless of whether the change occurred at a SLOW, MODERATE, FAST or VERY FAST rate (average (SD) and maximum CV among rates: 5.8% (3.2%) and 12.7%). Although pressure magnitudes may vary due to sensor drift, heat maps in **Figure 3B** show similar pressure distributions among test rates at various stages in the testing cycle. The measured pressure values during the VERY FAST test (which was performed first, and therefore should have accurate pressure readings) are within the range of values reported in the literature on pressures in tight-fitted sockets,^29^ showing local pressures of approximately 200 kPa. However, long-term sensor drift prevented direct, quantitative comparison of pressure magnitudes in this study and those in the literature. The extent of drift over a 24-hour period can be estimated by comparing peak pressures in **Figure 3B** between tests: the VERY FAST and MODERATE tests were performed 24 hours apart (VERY FAST and FAST were performed on the same day), as were the MODERATE and SLOW tests. In both cases, the observed drift was approximately 20% over 24 hours, underscoring its significance and the need to normalize pressure values.

### Repeatability and Hysteresis in Volume Adjustability

**Figure 4:**
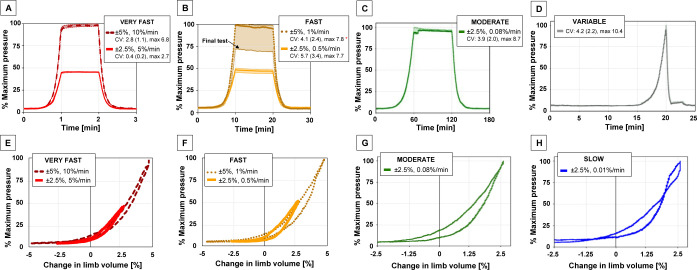
Repeatability of normalized pressure [%] vs. time [min], for **A**) VERY FAST, **B**) FAST, **C**) MODERATE and **D**) variable tests. Shaded regions show variation among three trials. CVs are reported as: average CV (standard deviation), maximum CV over the cycle, in [%]. Sample hysteresis curves of normalized pressure [%] vs. change in limb volume [%], for **E**) VERY FAST, **F**) FAST, **G**) MODERATE and **H**) SLOW tests. * Final test omitted in CV calculation.

Tests were repeatable at all flow rates (SLOW was not repeated due to long test times required) and the variable cycle test (**Figure 4A-D**). The largest CV of 10.4% (variable test, **Figure 4D**) corresponds to a pressure magnitude of approximately 6 kPa. For the variable test, pressure change was only detectable when approximately +1.5% volume change was achieved, therefore only the peak at 20 seconds and the hold at 21 seconds are visible in **Figure 4D**. Sensor malfunction occurred during the final test at the FAST rate for ±5% volume change, which is depicted in Figure 4B. Pressure measurements in **Figure 4A** and **4B** are normalized to the maximum pressure in the ±5% test.

**Figure 4E** and **4F** show similar hysteresis curves between ±2.5% and ±5% tests, verifying that limb volume changed in a similar fashion regardless of start and end volumes. The slight variation in pressure readings between ±2.5% and ±5% tests could be attributed to sensor drift or a minor difference in limb placement in the socket between tests. The relatively small hysteresis at each test rate (**Figure 4E-4H**) is confirmed by similar pressure distributions between points 1 and 5, and points 2 and 4 in **Figure 3B**. Any hysteresis could stem from hysteresis in the urethane, TPU bladder elasticity, or sensor drift.

### Symmetry Between Two Adjustable Areas

**Figure 5:**
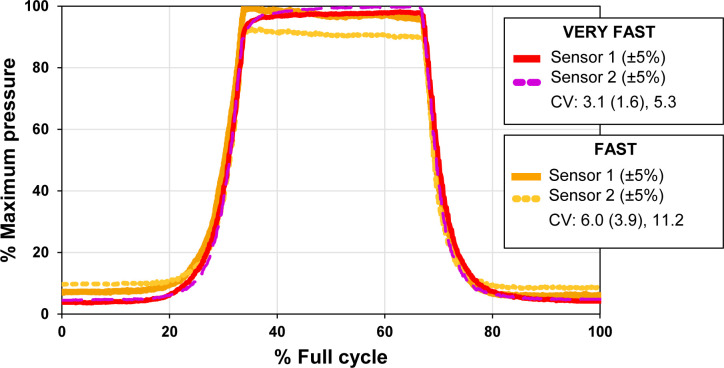
Normalized pressure [%] vs. cycle time [% full cycle] for sample VERY FAST and FAST tests (over ±5% volume change) measured at two bladder locations with two F-Socket sensors. CVs are reported as the average (standard deviation), maximum CV over the cycle, in [%].

Volume change symmetry was analyzed to identify potential manufacturing variances among the cavities or bladders. **Figure 5** shows the pressure over time for both sensor locations, illustrating very similar trends and pressure distributions. Small variations seen are expected due to the manual nature of the manufacturing process.

## DISCUSSION

The biofidelic limb enables a controlled, bench-top testing method for real-time evaluation of prosthetic sockets fitted to residual limbs experiencing volume fluctuations of up to ±5%. The value of bench-top testing has also been recognized by McGrath et al.,^14^ who developed a mock limb capable of simulating residual limb perspiration. Constructed from readily available materials for less than CAD 400, the biofidelic limb presented in this study offers many potential adaptations and alternative applications, as outlined in this Discussion. While the design met all predetermined design criteria, key study limitations are also addressed below.

### Potential Design Adaptations

In this study, specific materials and geometries were selected, but these can be adapted based on user needs. For example, a transfemoral limb model could have a greater diameter and height, with a thicker outer layer of the soft VytaFlex™ 20 urethane (or with urethane having a lower hardness and/or modulus) to better resemble the softer tissue often found in a transfemoral vs. transtibial residual limb. Additionally, a network of smaller bladder channels could be used to more closely resemble the flow of interstitial fluid within the limb. To promote replication and adaption, design files are provided on GitHub: https://github.com/Decisionics/BiofidelicLimb.

The deformation of the soft urethane (VytaFlexTM 20) surrounding the bladders is complex and was not investigated in this study. Indentation tests of the soft material used for the limb would help quantify its behaviour, as done by Rankin et al.,^12^ enabling a material-driven and performance-based design approach to optimize bladder placement and size, given the intended anatomical shape of the limb. Nonetheless, we expect that placing the bladders closer to the surface would produce a stiffer limb with a steeper pressure response, especially at smaller volumes.

The mandrel serves to mimic bone, but can also apply load for any load-bearing testing, which would, in general, increase interfacial pressure. For example, understanding effects on suspension from a fluctuating limb under load may be of interest, as it would expand on work by Quinlan et al.^24^ who previously evaluated the effects of textured socket surfaces on suspension using a static mock limb under load. McGrath et al.^14^ also evaluated their mock limb under loads that simulated the stance and swing phases of gait. Additionally, the rigid socket restricted limb expansion, and the stiffness of this socket affects the measured pressure distribution. Therefore, it is important to note the socket stiffness and applied load, especially if a rigid socket is used as a control comparison to an adjustable socket.

### Study Limitations

Our bladders were initially designed to accommodate a ±6.8% limb volume change, but achieved only ±5% due to nonlinear limb-bladder volume scaling, causing very little overall volume change with initial bladder expansion. This is likely due to the difference in compressibility between the soft outer urethane and the water-filled bladders. As the bladders expand from a small initial volume, the surrounding outer urethane is compressed. Then, once a certain bladder volume is reached, the surrounding urethane has stiffened enough that overall limb volume increases more proportionally with further bladder expansion. Further work is required to verify this hypothesis, including indentation tests to verify how the urethane stiffness varies as a function of bladder volume.

A key limitation of the study was that most tests were limited to ±2.5% volume change due to uncertainty in the heat-seal strength of the bladders at full expansion. While this volume range partially meets the target design criteria, the limited ±5% tests conducted still verified the biofidelic limb's functionality. Since results at ±2.5% and ±5% were comparable for the FAST and VERY FAST tests, it is reasonable to expect that the ±2.5% tests for SLOW and MODERATE can be generalized to the ±5% range. However, future designs should include a greater bladder capacity to ensure a safety margin, and the long-term bladder strength at full expansion, especially at heat-sealed joints, is a critical area for future evaluation.

The long-term use of the sensing system meant pressure magnitudes could not be reported or compared between tests; thus, pressures were normalized in the study to address this limitation. Furthermore, the sensor drift prevented any direct comparison of pressure magnitudes in this study to those in the literature. Despite this, using normalized pressures met the validation goals of the study because the trends, repeatability, and relative changes in pressure over the volume-change cycles remained consistent across repeated trials. This indicates that, even without pressure magnitudes, the system reliably captured the relation between changes in limb volume and interfacial pressure, which was an essential requirement for evaluating the limb's performance. For applications requiring absolute pressure, sensors should be calibrated before each test or replaced with new sensors following every test.

### Applications

The biofidelic limb can be used to evaluate sockets (both static and adjustable) fitted to residual limbs with volume fluctuations of up to ±5% in real time. Clinical outcomes of sockets (e.g., pistoning, suspension, and interfacial pressures) can be evaluated by adjusting the size of the limb, rather than swapping out sockets or limbs of different sizes.^2,25^ Furthermore, the detailed design description provided in this study expands on the work of Paterno et al.,^16^ who developed a similar limb but did not provide design details. The methods presented here could be combined with those from McGrath et al.^14^ to develop a biofidelic limb capable of both volume fluctuation and perspiration. Beyond prosthetics, the biofidelic limb may support testing the fit of footwear, compression garments or orthotics.^30,31^ Load-bearing testing is also facilitated via the internal mandrel.

## CONCLUSION

This work presents the design and fabrication procedure of a biofidelic limb used for testing prosthetic sockets, with adjustable water-filled bladders that enable controllable, repeatable volume changes of up to ±5% of limb volume. The biofidelic limb demonstrated high repeatability (CV < 10.4%) and adequate symmetry (CV < 11.2%) between two bladders placed on two areas of the limb. Despite its limitations, the sensing tool effectively confirmed the biofidelic limb's functionality.

The proposed design can be tailored for different anatomies, volume change ranges, materials, and is easily manufactured with readily available materials. Furthermore, it allows for controllable bench-top testing during prototyping of devices designed for volume adjustment, such as adjustable prosthetic sockets, eliminating the need for resource-intensive clinical trials.
